# Volcanic history in the Smythii basin based on SELENE radar observation

**DOI:** 10.1038/s41598-019-50296-9

**Published:** 2019-10-10

**Authors:** Ken Ishiyama, Atsushi Kumamoto

**Affiliations:** 1 National Institute of Technology, Tsuruoka College, 104 Sawada, Inooka, Tsuruoka, Yamagata, 997-8511 Japan; 2 Department of Geophysics, Graduate School of Science, Tohoku University, 6-3 Aramaki Aoba, Aoba-ku, Sendai, Miyagi 980-8578 Japan

**Keywords:** Rings and moons, Volcanology

## Abstract

Elucidation of the subsurface structure in the Smythii basin on the moon is important for understanding lunar volcanic history. Two lava units (Units 1 and 2) cover this basin. The spatial subsurface structure below Unit 2 is unknown. We used SELENE/Lunar Radar Sounder data to identify four subsurface boundaries at 130, 190, 300, and 420 m depths. The radar is reflected at the paleo-regolith layer sandwiched among lava flows, which is supported by a simple radar reflection/transmission model. The spatial distribution of subsurface boundaries demonstrates the deposition of Unit 2 on the subsidence in Unit 1. A simple loading model explained the maximum depth of subsidence (~500 m) and indicated that lithospheric thickness in the Smythii basin was ~24 km at 3.95 Gya. The estimated growth rate of the lithosphere was ~60 km/Ga during 3.95 to 3.07 Gya. After the formation of the Smythii basin at ~4.11 Gya, Unit 1 and Unit 2 deposited with eruption rates of ~8.4 × 10^−4^ km^3^/yr by 3.95 Gya and ~7.5 × 10^−6^ km^3^/yr by 3.07 Gya respectively. The timing of decline in volcanic activity in the Smythii basin differs from that for the lunar nearside maria, indicating the diversity of volcanism in various lunar areas.

## Introduction

Lunar volcanic history is crucial for understanding lunar thermal evolution^1–4^. After the formation of the moon ~4.5 Gya (i.e. gigayears ago), melt in the lunar magma ocean remained in the lunar mantle and affected the duration of lunar volcanic activity according to the lunar thermal evolution model^5,6^. During the late heavy bombardment period (~4.0–3.8 Gya)^7,8^, many basins formed on the lunar surface, and magma continued to erupt over a long time on the near side (especially in the Procellarum KREEP Terrane area) rather than the far side^3^.

**Figure 1:**
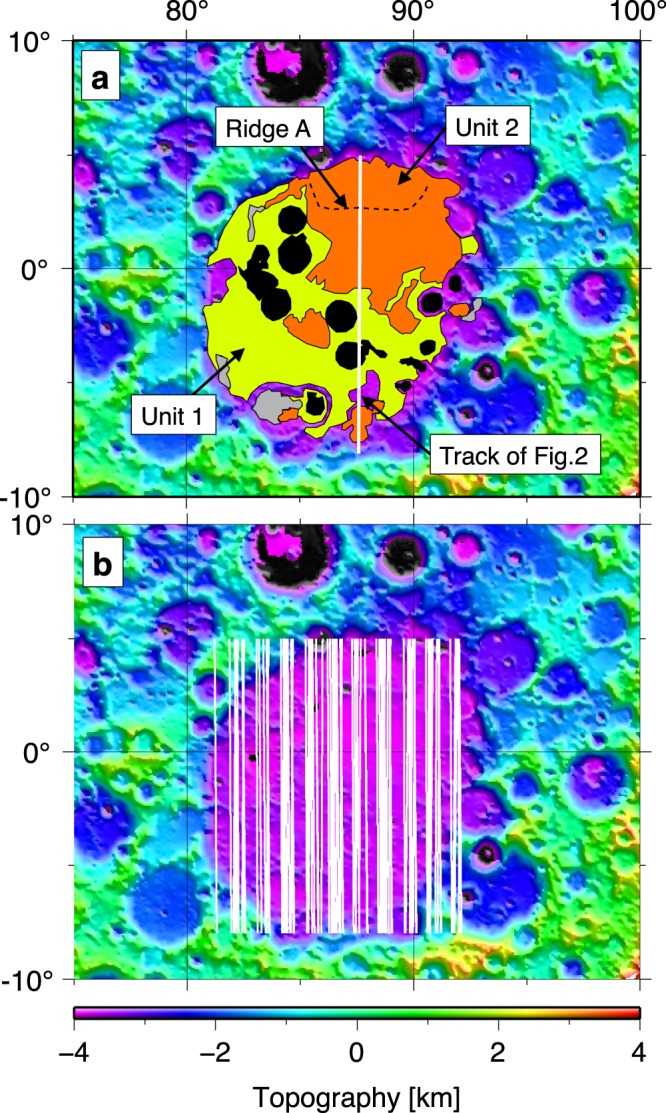
Topographical map of the Smythii basin. The background image shows lunar topography, which is created on the basis of SELENE/LALT data^35^. (**a**) Geological map^13^ based on the topographical map. The yellow area is Unit 1, the orange area is Unit 2, the grey area is dark mantle deposit, and the black area is where the distinction of units is difficult. The black dotted curve indicates the mare ridge (Ridge A in this study). The white line indicates the SELENE/LRS track used in Fig. 2. (**b**) Spatial distribution of LRS track data used in this study (white lines).

In this study, we focused on the Smythii basin (1°S, 87°E), located between the lunar near and far sides (Fig. 1a). This circular basin^9,10^ formed during the Pre-Nectarian era (before 3.92 Gya)^3^ and has five ring structures, which are ~130, 185, 270, 370, and 565 km in radius based on photogeologic mapping^11^. After basin formation, lava partially erupted and was deposited inside the basin, forming two mare units: Units 1 and 2 (Fig. 1a)^12,13^. The geological map of Fig. 1a has previously been published in the United States Geological Survey [https://pubs.er.usgs.gov/publication/i948].

Based on Apollo 15/X-ray data, Unit 1 is composed of early volcanic material, which is relatively older than that of Unit 2^12^. Unit 2, the young mare unit of the Smythii basin^3^, is located in the northeast part of the basin (Fig. 1a) and is composed of lava of uniform mineral composition (comprising FeO and TiO_2_)^14^. A ridge (hereafter “Ridge A”) following an east–west direction was formed on Unit 2. In general, lunar ridges are formed by a thermal decrease in lunar radius^15^ and by lava-flow loading^1,2^. Based on the crater chronology, the age of Unit 2 is 3.14 Ga (i.e. giga-annum or billion years)^3^, but the formation ages of Unit 1 and the Smythii basin have not yet been investigated clearly.

Based on the empirical relationship between the basin’s diameter and depth, the depth of the mare’s bottom (i.e. mare thickness) was estimated to be 1.28 km on average^16^. In addition, the ejecta composition of several craters on Unit 2 suggested that the mare thickness became shallower along the radial distance from the mare centre^17^.

Lunar subsurface structures have been investigated via seismic surveys and radar exploration^18,19^. The SELENE radar exploration detected one subsurface boundary at a depth of ~250 m in Unit 2^18^, and several subsurface boundaries below Unit 2 have also been reported^19^. However, the spatial distribution of these subsurface structures has not been investigated. The purpose of this study, therefore, is to identify subsurface boundaries in Unit 2 based on SELENE/Lunar Radar Sounder (LRS) data and to investigate the formation ages of the Smythii basin, Unit 1, and Unit 2. Based on these analyses, we provide suggestions regarding the volcanic history of the Smythii basin. The LRS data covered the area of 8.00°S–5.00°N and 81.00°E–92.00°E (Fig. 1b), which included 90 tracks.

## Results

**Figure 2:**
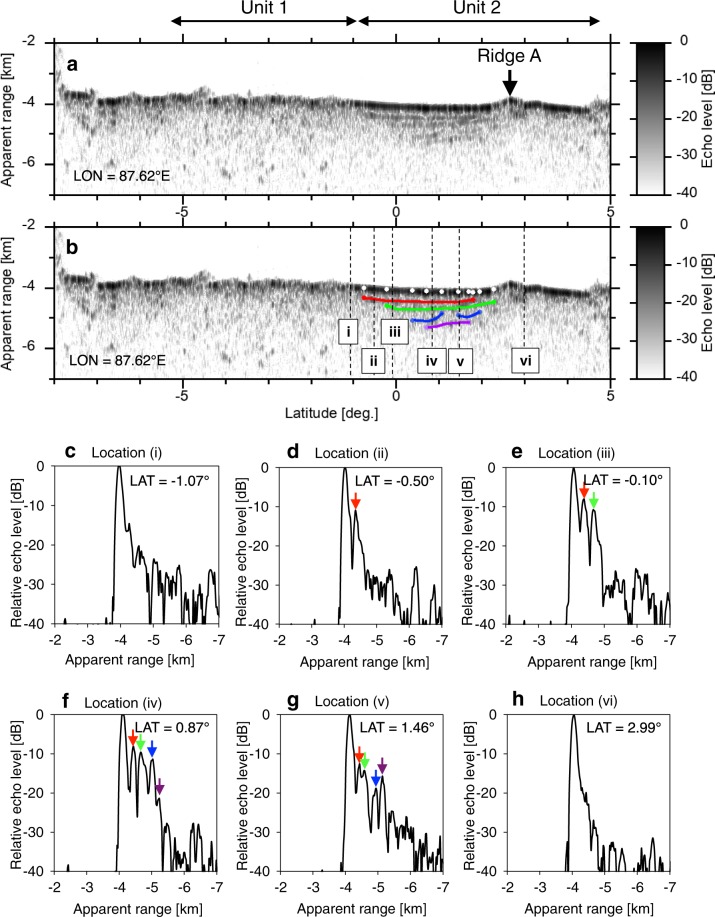
Typical radargram obtained from below Unit 2. This figure is created on the basis of SELENE/LRS data. (**a**) Radargram along 87.62°E (i.e. the white line in Fig. 1a). The colour bar shows the echo level, the horizontal arrows show the range of units, and the down arrow shows the location of Ridge A. (**b**) Radargram with traced subsurface echoes. The red, green, blue, and purple curves show subsurface boundaries 1–4, respectively. The white points on the surface echo indicate the location of the edges of four subsurface echoes, at which the depths of subsurface echo were measured in this study. The vertical dashed lines indicate locations (i–vi). (**c**–**h**) A-scope data at locations (i) (1.07°S), (ii) (0.50°S), (iii) (0.10°S), (iv) (0.87°N), (v) (1.46°N), and (vi) (2.99°N), respectively. The red, green, blue, and purple arrows show the echo peaks of subsurface boundaries 1–4.

**Figure 3:**
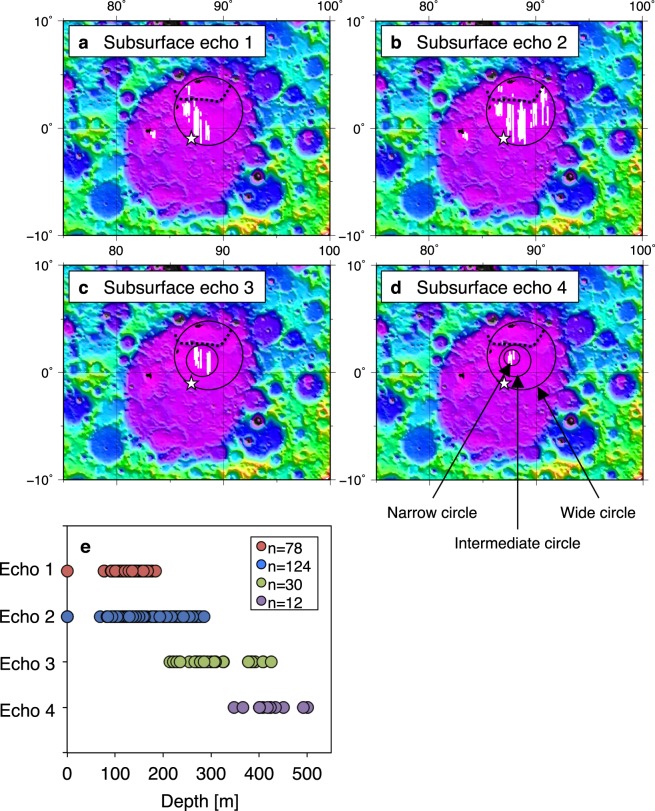
Spatial distribution of subsurface boundaries 1–4 ((**a**–**d**) respectively). The background image shows lunar topography, which is created on the basis of SELENE/LALT data^35^. The white lines show the locations of subsurface boundaries, the white star indicates the geological centre of the Smythii basin (1°S, 87°E), and the black dotted curve shows the location of Ridge A. The black circles (wide, intermediate, and narrow circles) show the areas of each subsurface echo. (**e**) Measured depth-distribution of subsurface echoes 1–4; “n” is the number of points at which surface and subsurface echoes was observed on a radargram.

Fig. 2a,b show the radargram obtained by the LRS data. Other radargrams and these tracks are also shown in Figs S1–S3. We identified four subsurface boundaries in Unit 2, namely subsurface boundaries 1–4. Fig. 2c–h show the echo intensity as a function of depth (i.e. A-scope plot) at locations i–vi, indicated in Fig. 2b. We could not confirm the subsurface echo at locations i and vi (Fig. 2c,h) but identified the clear peaks of subsurface echo in locations ii–v (Fig. 2d–g). Fig. 3a–d show the spatial distributions of the identified subsurface boundaries. The average depths of these subsurface boundaries are 130 ± 20, 190 ± 60, 300 ± 60, and 420 ± 50 m, respectively. Fig. 3e shows the range of the measured depth of each subsurface boundary. The deepest subsurface echo was ~500 m, located at 0.87°N, 87.41°E (i.e. within the narrowest circle in Fig. 3d). The shallow subsurface boundaries 1 and 2 are widely distributed in Unit 2 (Fig. 3a,b). These subsurface areas have a wide diameter of ~195 km, surface area of ~3.0 × 10^4^ km^2^, and centre at 1.60°N, 88.60°E (Table S1). The deep subsurface boundaries 3 and 4 are constrained within narrower areas inside the wide circles (Fig. 3c,d). The area of subsurface boundary 3 has an intermediate diameter of 90 km, surface area of 6.4 × 10^3^ km^2^, and centre at 1.10°N, 88.00°E. The area of subsurface boundary 4 has a narrow diameter of 45 km, surface area of 1.6 × 10^3^ km^2^, and centre at 1.40°N, 87.70°E. In addition, most subsurface boundaries were identified on the southern side of Ridge A. The volume of lava below Unit 2 was at least (6.6 ± 2.7) × 10^3^ km^3^, based on the simple multiplication of each layer’s thickness and each subsurface circle area.

**Figure 4:**
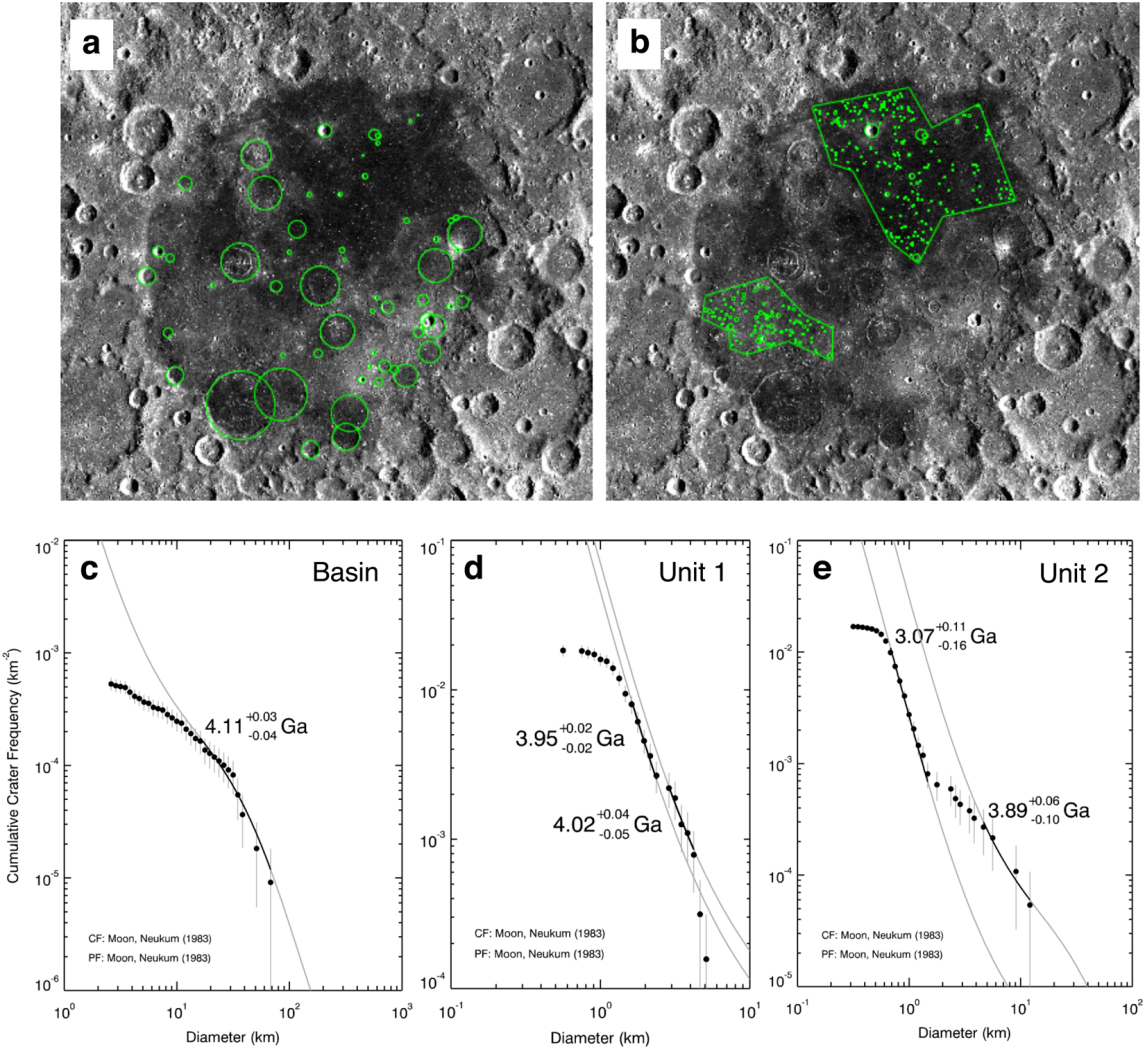
Measurement results of crater size-frequency distribution (CSFD) in Mare Smythii. (**a**) The analysed area of Smythii Basin. (**b**) The analysed areas of Units 1 and 2. The background image shows the ortho map, which is created on the basis of the SELENE/TC Ortho data^28^. The green circle shows the location of impact crater. (**c**) The CSFD of Smythii Basin. (**d**) The CSFD of Unit 1. (**e**) The CSFD of Unit 2. The grey curves are of the crater production function polynomial (i.e. PF)^29^. The black curves on the measured CSFD were fitted using the PF, and the suitable model ages were calculated using the cratering chronology function (i.e. CF)^29^.

Fig. 4a–e show the cumulative crater frequencies for the Smythii basin, Unit 1, and Unit 2. The investigated number of craters (Num) and surface areas (S) are respectively Num = 58 and S = 1.1 × 10^5^ km^2^ for Smythii basin, Num = 117 and S = 6.4 × 10^3^ km^2^ for Unit 1, and  Num = 314 and S = 1.9 × 10^4^ km^2^ for Unit 2. Based on the crater chronology (see Method), the model age of the Smythii basin is 4.11(+0.03/−0.04) Ga. The model age of Unit 1 is 3.95(+0.02/−0.02) Ga. An old buried surface aged 4.02(+0.04/−0.05) Ga exists under Unit 1. The model age of Unit 2 is 3.07(+0.11/−0.16) Ga, and an old buried surface aged 3.89(+0.06/−0.10) Ga exists beneath it. Therefore, after the formation of the Smythii basin at ~4.11 Gya, Unit 1 deposited by ~3.95 Gya, and Unit 2 deposited by ~3.07 Gya.

## Discussion

**Figure 5:**
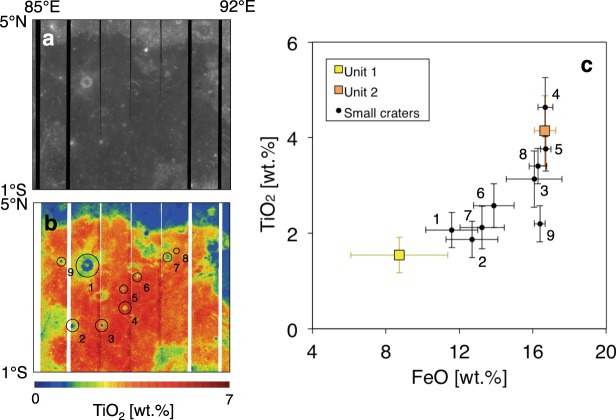
Mineral composition around small impact craters on Unit 2. This figure is created on the basis of the SELENE/Multiband Imager (MI) data. (**a**) 750 nm spectral data based on the MI data. (**b**) TiO_2_ map based on the MI data. This map data was produced on the basis of an algorism developed in a previous study^22^. The black circles show the locations of measured small craters in this study, and these are numbered in (**b**,**c**). (**c**) Comparison of mineral composition (TiO_2_ and FeO) among Unit 1 (yellow square), Unit 2 (orange square), and the small craters in Unit 2 (black point). Unit 1 is that TiO_2 _= 1.54 wt.% and FeO = 8.73 wt.%, and Unit 2 is that TiO_2_ = 4.15 wt.% and FeO = 16.66 wt.%.

We first discuss the depths of the subsurface boundaries and the cause of radar reflection. Ono *et al*.^18^ found one subsurface boundary below Unit 2 (~1.0°N, ~87.4°E), which corresponds to the subsurface boundary 2 in this study. The subsurface boundaries detected in this study are a few hundred metres deep and shallower than the total lava thickness in the Smythii basin (1.28 km)^16^; the LRS detected echoes not from the basin’s bottom but from a boundary between the lava layers. In addition, the ejecta composition around the small craters on Unit 2 (Fig. 5 and Table S2) shows an intermediate composition between Units 1 and 2, which indicates that Unit 2 deposited on Unit 1. Craters 4 and 5 locate around the centre of Unit 2, and these ejecta compositions are similar to the composition of Unit 2. This indicates that these craters do not excavate down to the depth of Unit 1; the boundary between Units 1 and 2 is deeper than ~420 m at the centre of Unit 2 according to the crater excavation depth (d_exc_ = 0.1 × 0.84 × Diameter)^20^ (Table S2), which is consistent with the depth of the deepest subsurface echo (~500 m).

Besides, we can expect that the boundary depth between Units 1 and 2 is relatively shallow on the north of Ridge A. For example, Craters 7 and 8 are located at the north of Ridge A, and the composition of Crater 7 is similar to that of Unit 1, while the composition of Crater 8 is similar to that of Unit 2. The excavation depths of these craters, namely ~210 m to ~320 m for Craters 7 and 8 (Table S2), can restrict the boundary depth between Units 1 and 2 around these craters. This is roughly consistent with the observation result that the shallow subsurface boundaries 1 and 2 slightly exist on the north of Ridge A but the deep subsurface boundaries 3 and 4 do not. We could not extensively determine the thickness of Unit 2 on the north of Ridge A. The reason for a small amount of shallow subsurface boundaries 1 and 2 on the north of Ridge A may be due to the non-uniform thickness of Unit 2 on the area.

In general, the radar reflection is caused by the permittivity contrast, which is derived from the existence of the regolith layer or the mineral composition contrast (TiO_2_ and FeO). In this study, we support the existence of the buried paleo-regolith layer under Unit 2 by using a simple radar reflection/transmission model (Method and Fig. S4). This model uses the following parameters: the contents of TiO_2_ and FeO in Units 1 and 2, porosity of Units 1 and 2 ($\varphi$), and time delay (i.e. round-trip time) between the surface and subsurface echo (Δ*t*). The surface mineral compositions are TiO_2_ = 1.54 wt.% and FeO = 8.73 wt.% in Unit 1, and TiO_2_ = 4.15 wt.% and FeO = 16.66 wt.% in Unit 2 as per the analysis of SELENE/Multiband Imager data^21,22^. Based on these compositions, the estimated bulk permittivity values of Units 1 and 2 are 6.61 and 7.73, respectively. Assuming that $\varphi$ = 7% based on the Apollo sample^23^ and Δ*t* = 3.33 × 10^−6^ s based on the depth of the deepest subsurface echo (~500 m), the estimated level of the deepest subsurface echo was about −35 dB with respect to the level of surface echo. As shown in Fig. 2, the LRS mainly detected the strong subsurface echoes of −20 dB or more; the mineral composition contrast between Units 1 and 2 cannot explain the observed radar reflection. If a regolith layer exists under Unit 2, the estimated level is about −17 dB, which is close to the level of the deepest subsurface echo (Fig. 2).

The existence of the buried paleo-regolith layer suggests that the lava of Unit 2 was discretely erupted and deposited. Since there are four subsurface boundaries under Unit 2, the lavas erupted at least five separate times. If the lunar lava deposited quickly, the average pause interval between lava eruptions for Unit 2 is ~0.18 Ga, which was simply calculated by dividing the formation period of Unit 2 (3.95 to 3.07 Ga) by 5. Thus, this pause interval can contribute to the development of the paleo-regolith layer. After the formation of the Smythii basin at ~4.11 Gya, the lava of Unit 1 finished depositing in a short time (by ~3.95 Gya); this rapid deposition may explain why the LRS cannot identify the subsurface echoes under Unit 1.

Next, we focused on the spatial distribution of the subsurface boundaries in Unit 2 (Fig. 3). Mare Smythii has semi-major and -minor axes (~420 and ~330 km, respectively), and a surface area of 1.1 × 10^5^ km^2^ (Table S1). The shallow boundaries 1 and 2 lie within the wide circle. The spatial distribution of subsurface boundary 1 is relatively narrower than that of subsurface boundary 2, which indicates that Unit 2 is composed of at least two different subunits. The subsurface boundaries 3 and 4 narrowly distribute within the wide circle. The deepest location of the subsurface boundary was 0.87°N, 87.41°E (i.e. within the smallest circle), which differs from the geological centre of Mare Smythii (1.00°S, 87.00°E) (see the star in Fig. 3). The spatial structure based on the subsurface circles in Fig. 3 suggests that Unit 2 deposited on a ground subsidence on Unit 1, because the all-subsurface layers are deposited in parallel and the edges of the strata fade with depth, giving the appearance of bowls. We inferred that this subsidence was produced by the loading deflection of Unit 1.

Using a simple loading model^1^ (see Method), for example, suppose that Unit 1, with a radius of ~100 km (i.e. load), deposited on the lithosphere of thickness (T_litho_ = ~24 km) when Unit 1 formed (~3.95 Gya). Then, we can explain the observed subsidence depth (~500 m). This lithospheric thickness is consistent with the result of Solomon and Head^2^; the lithospheric thickness grows with time. The growth rate of the lithosphere in the Smythii basin is higher than ~60 km/Ga during 3.95 to 3.0 Gya, based on the results of this study (T_litho_ = 24 km at 3.95 Gya) and those of Solomon and Head^2^ (T_litho_ ≥ 75 km at ~3.0 Gya). On the other hand, lunar thermal evolution model showed that the growth rate was ~75 km/Ga during 3.95 to 3.0 Gya based on the result of Spohn *et al*.^5^ (e.g., the curve showing 1073 K in their Fig. 2b), which is consistent with the result for the Smythii basin.

Finally, we discuss the volume and eruption rate of lava in the Smythii basin. The total thickness of the lava in the Smythii basin was 1.28 km^16^ and the surface area of Mare Smythii was 1.1 × 10^5^ km^2^. Thus, the total volume of lava in Mare Smythii was ~1.4 × 10^5^ km^3^, which is much higher than the volume of Unit 2 (6.6 × 10^3^ km^3^) (Table S2); the Smythii basin is almost covered by the lava of Unit 1 (~1.3 × 10^5^ km^3^). The estimated eruption rate of the lava in Unit 1 was ~8.4 × 10^−4^ km^3^/yr during 4.11 to 3.95 Gya, which is close to the average rate corresponding to the lunar near side maria (~10^−3^ to ~10^−5^ km^3^/yr)^24^. This indicates that the volcanic activity in the Smythii basin was extremely intensive before 3.95 Gya. Thereafter, at least 6.6 × 10^3^ km^3^ of lava erupted in Unit 2 by 3.07 Gya, the eruption rate being ~7.5 × 10^−6^ km^3^/yr during 3.95 to 3.07 Gya, which is lower than the average rate for the lunar near side maria.

The eruption rate rapidly decreased after ~3.4 Gya in the lunar nearside maria^24^, but after 3.95 Gya in the Smythii basin; the timing of decline in volcanic activity differs between the lunar nearside maria and the Smythii basin. The low eruption rate was caused by the increase in lithospheric thickness owing to lunar thermal cooling^2^ or by the depletion of the lava source in the lunar mantle^5^. Therefore, the state of lunar thermal evolution probably differs among the lunar nearside, farside, and boundary between the two hemispheres (such as the location of the Smythii basin). If lunar thermal cooling was dominant, the growth of the lithosphere after 3.95 Gya (>~60 km/Ga) may have prevented the eruption of lava. A comprehensive understanding of volcanic history in various areas would shed light on the complexities of lunar thermal evolution.

## Methods

### Average depths of subsurface boundaries 1 to 4

We used the LRS data to investigate the subsurface structures in the Smythii basin. The LRS is an observation instrument that performs global subsurface radar sounding^25^ and transmits electromagnetic waves of 4–6 MHz at an interval of 75 m along the SELENE orbital track from an altitude of ~100 km from the lunar surface. It measures the time delay difference between the surface and subsurface echoes (Δ*t*)^25^. The LRS data cover the area spanning 8°S–5°N to 81°E–92°E (Fig. 1b), which includes 90 tracks. To eliminate clutter noise caused by lunar surface topography, we used synthetic aperture radar (SAR)-processed LRS data^26^ [http://darts.isas.jaxa.jp/planet/pdap/selene/index.html.en]. The elimination capability of noise was shown by Kobayashi *et al*.^26^ (e.g., see their Figs 8 and 9). The synthetic aperture is 5 km, and the spatial resolution is ~0.8 km along the orbital track direction and ~4 km along the longitude direction. Based on the analysis of Ono *et al*.^18^, we identified four subsurface boundaries from the continuity of the subsurface echo along the latitude, and confirmed the existence of subsurface echoes between adjacent orbits.

The depth of the subsurface boundary at a local location (${d}_{{\varepsilon }_{{\rm{bulk}}}}$) is given by

1 $${d}_{{\varepsilon }_{{\rm{bulk}}}}=\frac{c}{\sqrt{{\varepsilon }_{{\rm{bulk}}}}}\cdot \frac{\Delta t}{2}=\frac{{d}_{{\rm{radar}}}}{\sqrt{{\varepsilon }_{{\rm{bulk}}}}},$$

where *c* is the speed of electromagnetic wave in a vacuum (3 × 10^8^ m/s), ${\varepsilon }_{{\rm{bulk}}}$ is the bulk permittivity of the subsurface layer, and *d*_radar_ is the apparent radar depth, which is calculated by assuming that ${\varepsilon }_{{\rm{bulk}}}$ is the same as that in a vacuum (${\varepsilon }_{{\rm{bulk}}}=1$). The bulk permittivity of lunar basalt is 4–11 based on the Apollo basalt samples^27^, mode value of which is 6 to 7 in permittivity histogram. In this study, we supposed that ${\varepsilon }_{{\rm{bulk}}}=6$, so the depth at local location (*d*_*6*_) is given as

2 $${d}_{6}=\frac{{d}_{{\rm{radar}}}}{\sqrt{6}}.$$

If the standard deviation of permittivity is ±5, the subsurface boundary depth has the standard deviation of ~17% with reference to *d*_radar_.

We measured the depths (*d*_*6*_) of both ends of the continuous subsurface boundary along the latitude on the radargram. For example, the measured locations are shown on the surface echo (see the 10 white points on the surface echo shown in Fig. 2b). Subsequently, we estimated the average depth for each subsurface boundary (*d*_ave_) as

3 $${d}_{{\rm{ave}}}=\frac{\sum \,{d}_{6}}{N},$$

where *N* is the total number of measured locations for a subsurface boundary, and $\sum \,{d}_{6}$ is the summation of all average subsurface boundary depths. However, there are caveats in the calculation of Eq. 4. For example, we identified the outcrop of the subsurface boundary, edge depth of which is 0 m. We excluded the zero depth when using Eq. 3. The error of subsurface boundary depth was simply obtained from the standard deviation of the data set used in Eq. 3.

### Crater chronology

To determine the surface ages of the Smythii basin, Unit 1, and Unit 2 based on the crater chronology, we first measured the crater size–frequency distribution (CSFD) for Unit 1. The CSFD was obtained using the SELENE/TC Ortho map data^28^ (Fig. 4) [http://darts.isas.jaxa.jp/planet/pdap/selene/index.html.en]. These data were resampled to the spatial resolution of ~74 m/pixel. Subsequently, we fitted the measured CSFD to the crater production function (Eq. 4)^4^:

4 $${\log }_{10}\,N(D)={a}_{0}+\mathop{\sum }\limits_{k=1}^{11}\,{a}_{k}\,{({\log }_{10}D)}^{k},$$

where *D* is the crater diameter [km], *N*(*D*) is the cumulative number of craters per km^2^ larger than *D*, and *a*_0_ and *a*_n_ are the coefficients based on the study by Neukum^29^. Thus, *N*(1), that is, the cumulative number of craters on the grey curve of 10^0^ km diameter (e.g. Fig. 4e), is obtained. Finally, we estimated the surface age of Unit 1 (*t* [Ga]) by substituting *N*(1) in the crater chronology function (Eq. 5)^4^:

5 $$N(1)=A\,\{\exp \,(Bt)-1\}+Ct,$$

where *A*, *B*, and *C* are the coefficients based on the study by Neukum^29^. To make these calculations smooth, we used Craterstats 2.0 software^30^. The error of surface age was based on the definition of previous study^3^.

### Radar reflection/transmission model

We analytically calculated the echo intensity using a simple radar reflection/transmission model^25^. For example, we considered three layers (i.e., vacuum, Unit 1, and Unit 2) (Fig. S4). The intensity ratio of the surface and subsurface echoes is given as

6 $$\frac{{P}_{{\rm{subsurface}}}}{{P}_{{\rm{surface}}}}\approx \frac{{R}_{2,1}{(1-{R}_{0,2})}^{2}}{{R}_{0,2}}\,\exp \,(\,-\,\Delta t\cdot 2\pi f\cdot \,\tan \,\delta ),$$

where *R*_0,2_, *R*_2,1_, and tan *δ* are as follows:

7 $${R}_{0,2}={(\frac{\sqrt{{\varepsilon }_{{\rm{bulk}}\_0}}-\sqrt{{\varepsilon }_{{\rm{bulk}}\_2}}}{\sqrt{{\varepsilon }_{{\rm{bulk}}\_0}}+\sqrt{{\varepsilon }_{{\rm{bulk}}\_2}}})}^{2},$$

8 $${R}_{2,1}={(\frac{\sqrt{{\varepsilon }_{{\rm{bulk}}\_2}}-\sqrt{{\varepsilon }_{{\rm{bulk}}\_1}}}{\sqrt{{\varepsilon }_{{\rm{bulk}}\_2}}+\sqrt{{\varepsilon }_{{\rm{bulk}}\_1}}})}^{2},$$

and

9 $$\tan \,\delta ={10}^{0.045\times C-2.754}.$$

*R*_0,2_ and *R*_2,1_ are reflection coefficients on the boundaries between the vacuum and Unit 2 and between Unit 2 and Unit 1 respectively, Δ*t* is the time delay between the surface and subsurface echoes, *f* is frequency (5 MHz), tan *δ* is the loss tangent of Unit 2, and *C* is the mineral composition (TiO_2_ + FeO [wt.%]). ${\varepsilon }_{{\rm{bulk}}\_0}$ is the permittivity of vacuum (${\varepsilon }_{{\rm{bulk}}\_0}$ = 1). ${\varepsilon }_{{\rm{bulk}}\_1}$ and ${\varepsilon }_{{\rm{bulk}}\_2}$ are the bulk permittivities of Units 1 and 2, which are approximated on the basis of the effective medium theory^31^:

10 $${\varepsilon }_{{\rm{bulk}}}={\varepsilon }_{{\rm{grain}}}+3{\varepsilon }_{{\rm{grain}}}\frac{b}{b-1},$$

and

11 $$b=\varphi \frac{1-{\varepsilon }_{{\rm{grain}}}}{1+2{\varepsilon }_{{\rm{grain}}}}.$$

*ϕ* is the porosity of the subsurface layer, and ${\varepsilon }_{{\rm{grain}}}$ is pore-free permittivity (i.e. grain permittivity) of the subsurface layer^32,33^.

12 $${\varepsilon }_{{\rm{grain}}}={1.919}^{{\rho }_{{\rm{grain}}}},$$

where ${\rho }_{{\rm{grain}}}$ is given as

13 $${\rho }_{{\rm{g}}{\rm{r}}{\rm{a}}{\rm{i}}{\rm{n}}}=0.0273\cdot FeO+0.0110\cdot Ti{O}_{2}+\mathrm{2.773.}$$

When the mineral compositions are such that TiO_2_ = 1.54 wt.% and FeO = 8.73 wt.% for Unit 1and TiO_2_ = 4.15 wt.% and FeO = 16.66 wt.% for Unit 2 (Fig. 5) and *ϕ* = 7% based on the Apollo sample^23^, ε_bulk_1_ is 6.27 and ε_bulk_2_ is 7.27. If Δ*t* = 3.33 × 10^−6^ s based on the depth of the deepest subsurface echo (~500 m), the level of the subsurface echo is about −35 dB with respect to level of the surface echo as per Eq. 6. Considering that the permittivity of Unit 1 is equal to that of the regolith (${\varepsilon }_{{\rm{bulk}}\_1}=2$), we can estimate that the echo level is about −17 dB using Eq. 6.

### Loading model

We analytically calculated the subsidence of Unit 1 based on a simple loading model^1^. For example, we supposed a lava load (i.e. Unit 1) on the lithosphere (Fig. S5). In this study, the load has a radius *r* of 97.5 km with reference to the radius of Unit 2, a thickness *h* of 1.28 km, and bulk density ${\rho }_{{\rm{unit}}1}$, and the lithosphere has a thickness T_litho_ of 24 km. The subsidence (*w*) is given by the basic partial differential equation^34^.

14 $${\nabla }^{4}w+\frac{w}{{\ell }^{4}}=\frac{q}{A},$$

where *q* is the load and $\ell$ is the relative stiffness of the lithosphere.

15 $$q={\rho }_{{\rm{unit}}1}\cdot g\cdot h,$$

16 $${\ell }^{4}=\frac{A}{B},$$

17 $$A=\frac{E\cdot {T}_{{\rm{litho}}}^{3}}{12(1-{\nu }^{2})},$$

and

18 $$B=\frac{E\cdot {T}_{{\rm{litho}}}}{{R}^{2}}+{\rho }_{m}\cdot g.$$

*E* is Young’s modulus (=100 GPa), *ν* is Poisson’s ratio (=0.25), *R* is the lunar radius (=1737.4 km), ${\rho }_{{\rm{unit}}1}$ is given as ${\rho }_{{\rm{grain}}}(1-\varphi )$ based on Eq. 13 and mineral composition of Unit 1, ${\rho }_{m}$ is the bulk density of the lunar mantle (=3400 kg m^−3^), and *g* is lunar gravity (=1.64 m s^−2^). If *ϕ* = 7%, TiO_2_ = 1.54 wt.%, and FeO = 8.73 wt.% in Unit 1, *ρ*_until1_ = ~2800 kg m^−3^. The solution of Eq. 14 is approximately given as the function of the distance from the load’s centre (*x*, dimensionless parameter):

19 $${w}_{{\rm{in}}}(x)=\frac{q\cdot a}{B}({\rm{Ker}}^{\prime} a\cdot {\rm{Ber}}\,x-{\rm{Kei}}^{\prime} a\cdot {\rm{Bei}}\,x+1/a)\,(x\le a),$$

and

20 $${w}_{{\rm{out}}}(x)=\frac{q\cdot a}{B}({\rm{Ber}}^{\prime} a\cdot {\rm{Ker}}\,x-{\rm{Bei}}^{\prime} a\cdot {\rm{Kei}}\,x)\,(x\ge a),$$

where *w*_in_ and *w*_out_ are subsidence inside and outside the load; Ker, Kei, Ber, and Bei are the Bessel–Kelvin functions of order zero; and the prime denotes the first derivative^1^. *a* is the load radius normalized by $\ell$.

21 $$a=\frac{r}{\ell }.$$

When *x* is 0, the maximum depth of subsidence is given by Eq. 19:

22 $${w}_{{\rm{in}}}(0)=\frac{q\cdot a}{B}({\rm{Ker}}^{\prime} a+1/a),$$

where ${\rm{Ber}}\,(0)=1$, and ${\rm{Bei}}\,(0)=0$. In this study, *a* = 1.47 from Eq. 21 and ${\rm{Ker}}^{\prime} (1.45)\approx -\,3.10$. Thus, ${w}_{{\rm{in}}}\,(0)=502\,{\rm{m}}$, which is consistent with the depth of deepest subsurface echo (~500 m) based on the LRS observation.

## Supplementary information

Supplementary Figures and Tables
