# Supplementary material for: Volcanic history in the Smythii basin based on SELENE radar observation
Source: Sci Rep. 2019 Oct 10;9:14502. doi: 10.1038/s41598-019-50296-9 (PMC6787070; doi:10.1038/s41598-019-50296-9)
Supplement: Supplementary file 1 — Supplementary Figures and Tables [file 41598_2019_50296_MOESM1_ESM.docx]

**Volcanic history in the Smythii basin based on SELENE radar observation**

Ken Ishiyama^＊^ (corresponding author)

Atsushi Kumamoto

**
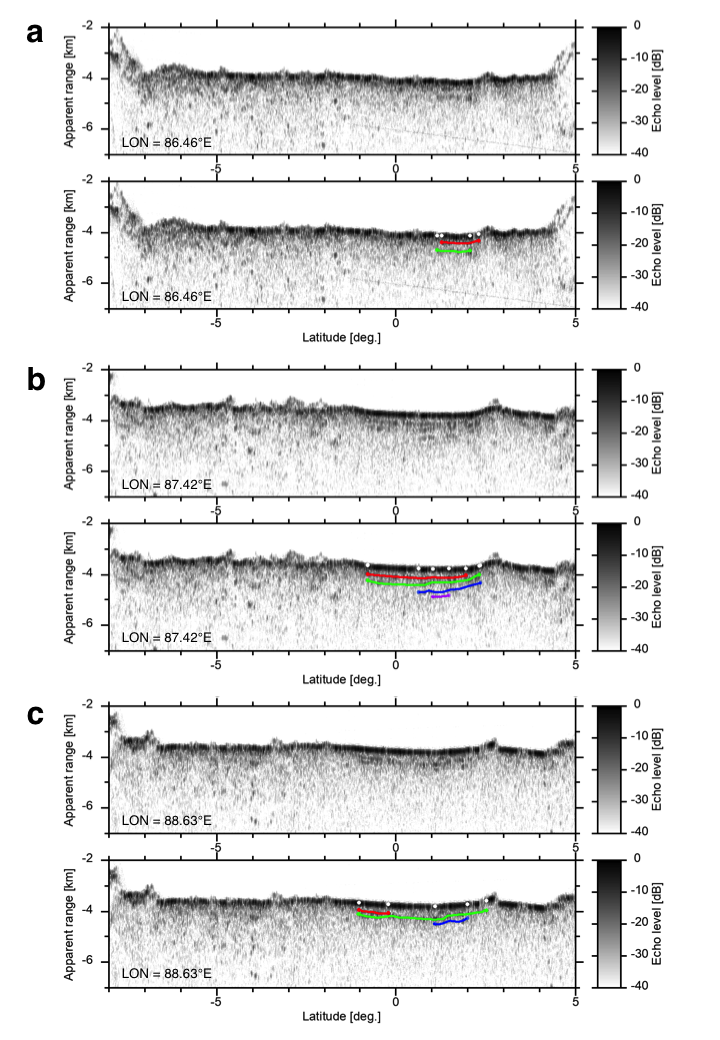
**

**Fig. S1 Radargrams based on the SELENE/LRS data.** (a) Track of 86.46°E. (b) Track of 87.42°E. (c) Track of 88.63°E.

**
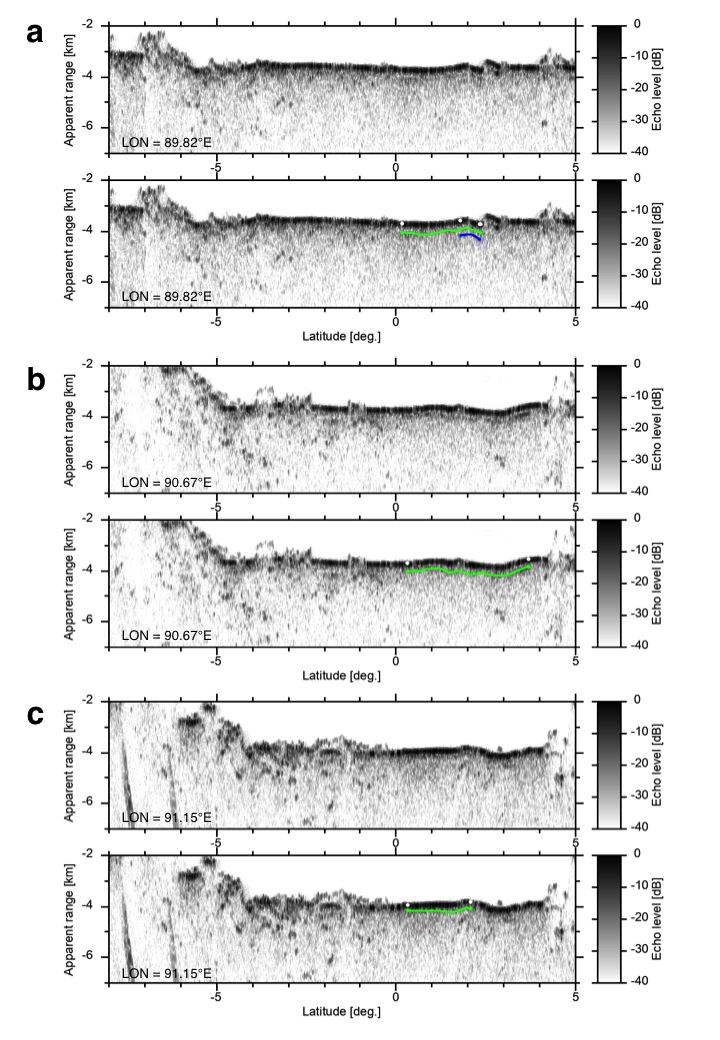
**

**Fig. S2 Radargrams based on the SELENE/LRS data.** (a) Track of 89.82°E. (b) Track of 90.67°E. (c) Track of 91.15°E.

**
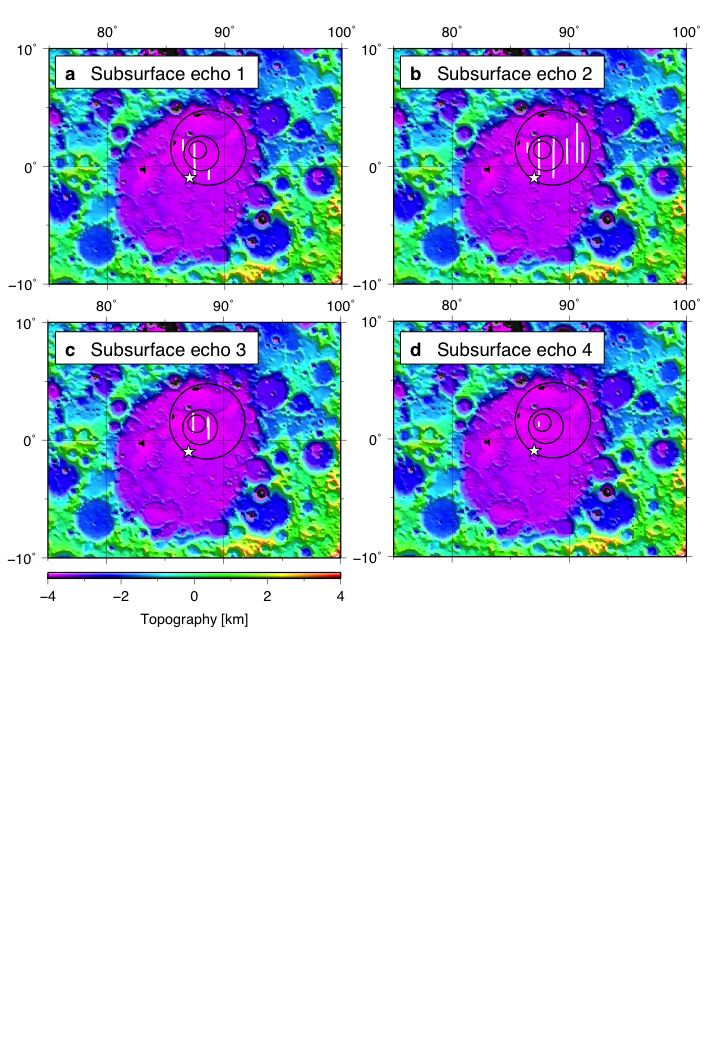
**

**Fig. S3 Spatial distribution of subsurface boundaries 1–4 based on the tracks of Figs. S1 and S2.** (a) Subsurface echo 1, (b) Subsurface echo 2, (c) Subsurface echo 3, and (4) Subsurface echo 4. The black circles show the area of each subsurface echo. The white star the geological center of the Smythii basin (1°S, 87°E).

**
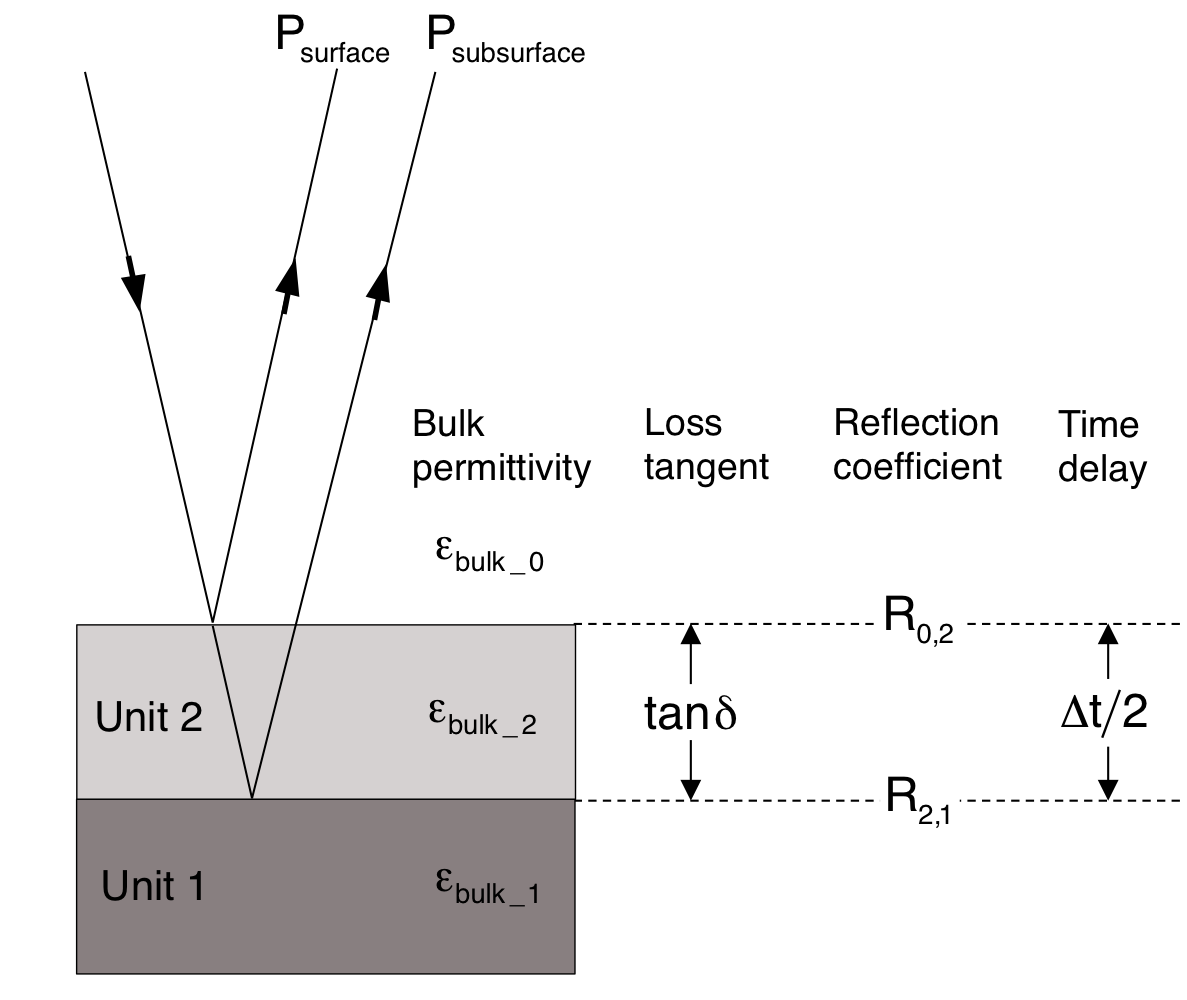
**

**Fig. S4 Schematic image of simple radar reflection/transmission model.** Unit 2 deposits on Unit 1, and the electromagnetic wave is reflected at surface of Unit 2 and at the boundary between Unit 2 and 1.

**
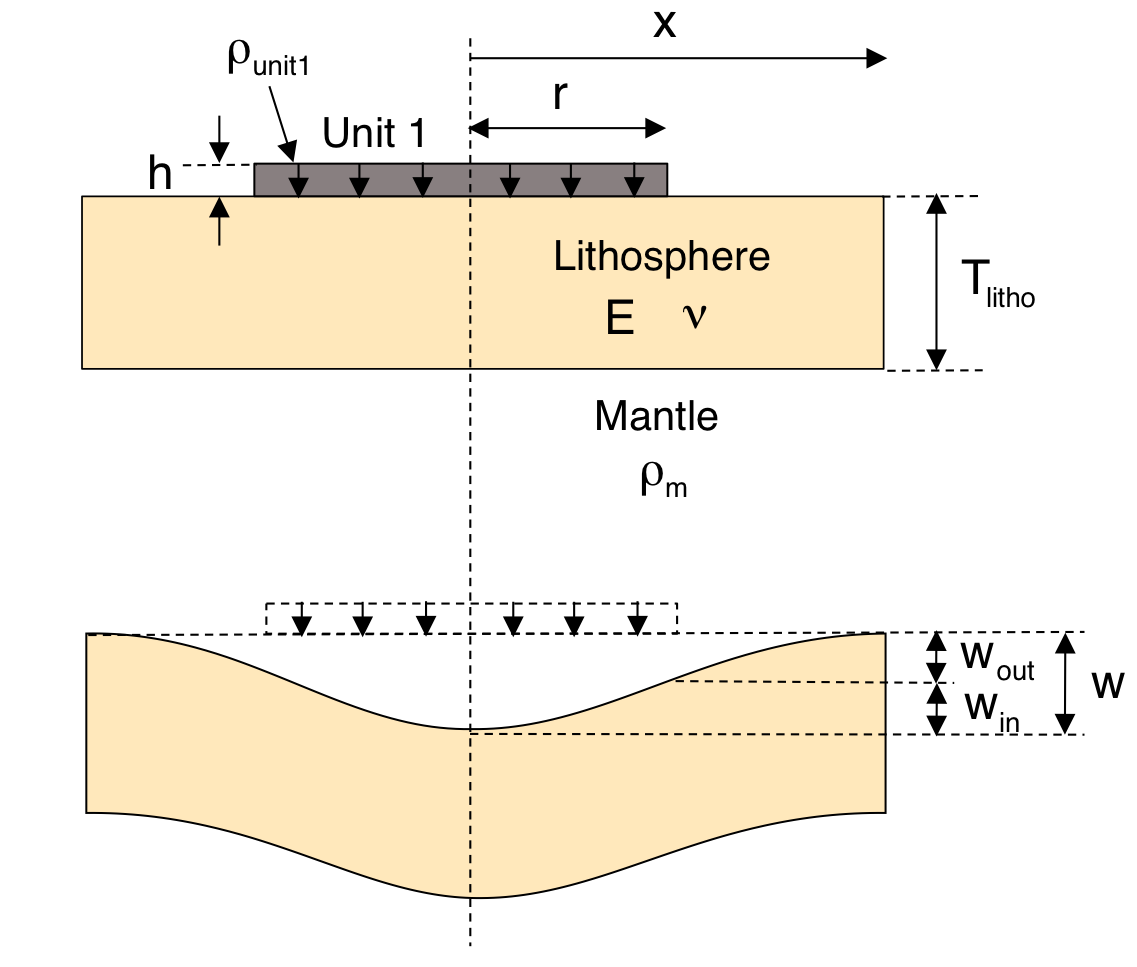
**

**Fig. S5 Schematic image of simple loading model.** The condition before subsidence. Unit 1 deposits on lithosphere, which is bent by the load of Unit 1.

Table S1 Summary of Mare Smythii, Unit 1, and Unit 2.

|  | Diameter [km] | Surface area [km^2^] | Thickness [km] | Volume [km^3^] |
| --- | --- | --- | --- | --- |
| Mare Smythii | - | 1.1×10^5^ | 1.28 | 1.4×10^5^ |
|  |  |  |  |  |
| Unit 2 (Wide circle) | 195 | 3.0×10^4^ | 0.19±0.06 | (5.7±1.8)×10^3^ |
| Unit 2 (Intermidiate circle) | 90 | 6.4×10^3^ | 0.11±0.12 | (7.0±7.6)×10^2^ |
| Unit 2 (Narrow circle) | 45 | 1.6×10^3^ | 0.12±0.11 | (1.9±1.8)×10^2^ |
| Unit 2 (Total) | - | - | 0.42±0.05 | (6.6±2.7)×10^3^ |
|  |  |  |  |  |
| Unit 1 | - | 7.9×10^4^ | - | (1.3±2.2)×10^5^ |

The thickness of Mare Smythii was based on the result of Williams and Zuber ^16^.

The surface area of Unit 2 was treated as the surface area of the wide circle.

The volume of Unit 2 was calculated from the summation of volume of each circle.

The surface area of Unit 1 was not taken into account the area hidden under Unit 2.

The volume of Unit 1 was calculated by subtracting the total volume of Unit 2 from Mare Smythii.

Table S2 Summary of parameters of small craters on Unit 2.

| Crater number | TiO_2_ [wt.%] | σ_TiO2_ [wt.%] | FeO [wt.%] | σ_FeO_ [wt.%] | Diameter [km] | Excavation depth [m] |
| --- | --- | --- | --- | --- | --- | --- |
| 1 | 2.07 | 0.37 | 11.59 | 1.42 | 12.7 | 1070 |
| 2 | 1.87 | 0.38 | 12.68 | 1.41 | 3.8 | 317 |
| 3 | 3.13 | 0.59 | 16.07 | 1.52 | 3.5 | 290 |
| 4 | 4.64 | 0.62 | 16.68 | 0.41 | 5.0 | 417 |
| 5 | 3.76 | 0.46 | 16.70 | 0.29 | 2.7 | 230 |
| 6 | 2.58 | 0.46 | 13.88 | 1.10 | 3.0 | 255 |
| 7 | 2.12 | 0.45 | 13.23 | 1.21 | 3.8 | 323 |
| 8 | 3.40 | 0.37 | 16.27 | 0.48 | 2.4 | 205 |
| 9 | 2.20 | 0.38 | 16.38 | 0.29 | 1.1 | 90 |

σ shows the standard deviation of titanium or iron.
